# Contributions to the Diagnosis and Treatment of Spinal Neuroses

**Published:** 1846-01

**Authors:** 


Art. VIII.—*Beiträge zur Erkenntniss und Heilung der Spinal-Neurosen.* Von Dr. Georg Hirsch in Königsberg.—*Königsberg,* 1843.

*Contributions to the Diagnosis and Treatment of Spinal Neuroses.* By Dr. G. Hirsch, &c. *Königsberg,* 1843. 8vo, pp. 466.

This is an elaborate monograph on what has been usually termed spinal irritation. It is characterized by the research and minuteness usually found in German works, and will form a good book of reference, although the author has not made himself acquainted with all the views that have been broached on the subject. He confines himself to the old beaten track through the *anatomical* relations of the spinal cord, and omits to notice the more recent views propounded by Dr. Laycock as to the influence of the physiological relations of organs, or, in other words, their sympathies in determining the organ, or class of organs, in which the phenomena of spinal irritation appear. The consequence is, that the higher or cerebral phenomena of hysteria, and spinal irritation, as insane secretiveness, abhorrence or love of colours, and the like, find no place in his Contributions. Yet it is certain that these are analogous in their origin to the symptoms which indicate irritation of the spinal cord only. As, however, we have, on more than one occasion, lately brought this subject before our readers, we must content ourselves with this hint to Dr. Hirsch, and others of his class, as to the propriety of adopting more comprehensive principles in laying down their pathology. It may be convenient for a systematic arrangement to distinguish the symptoms of spinal affections from those of cerebral affections, just as it is convenient to distinguish one artery from another. But when the general pathology of the nerves, or vascular system, is under discussion, it is well to get rid of these mere aids to arrangement, and let the mind take in the whole, unembarrassed by details of minor and sometimes doubtful value.

